# Unexpected Diversity of Feral Genetically Modified Oilseed Rape (*Brassica napus* L.) Despite a Cultivation and Import Ban in Switzerland

**DOI:** 10.1371/journal.pone.0114477

**Published:** 2014-12-02

**Authors:** Juerg Schulze, Tina Frauenknecht, Peter Brodmann, Claudia Bagutti

**Affiliations:** State Laboratory Basel-City, Biosafety Laboratory, Basel, Switzerland; University of Western Sydney, Australia

## Abstract

Despite cultivation and seed import bans of genetically modified (GM) oilseed rape (*Brassica napus* L.), feral GM plants were found growing along railway lines and in port areas at four sites in Switzerland in 2011 and 2012. All GM plants were identified as glyphosate-resistant GM event GT73 (Roundup Ready, Monsanto). The most affected sites were the Rhine port of Basel and the St. Johann freight railway station in Basel. To assess the distribution and intra- and interspecific outcrossing of GM oilseed rape in more detail, we monitored these two sites in 2013. Leaves and seed pods of feral oilseed rape plants, their possible hybridization partners and putative hybrid plants were sampled in monthly intervals and analysed for the presence of transgenes by real-time PCR. Using flow cytometry, we measured DNA contents of cell nuclei to confirm putative hybrids. In total, 2787 plants were sampled. The presence of GT73 oilseed rape could be confirmed at all previously documented sampling locations and was additionally detected at one new sampling location within the Rhine port. Furthermore, we found the glufosinate-resistant GM events MS8xRF3, MS8 and RF3 (all traded as InVigor, Bayer) at five sampling locations in the Rhine port. To our knowledge, this is the first time that feral MS8xRF3, MS8 or RF3 plants were detected in Europe. Real-time PCR analyses of seeds showed outcrossing of GT73 into two non-GM oilseed rape plants, but no outcrossing of transgenes into related wild species was observed. We found no hybrids between oilseed rape and related species. GM plants most frequently occurred at unloading sites for ships, indicating that ship cargo traffic is the main entry pathway for GM oilseed rape. In the future, it will be of major interest to determine the source of GM oilseed rape seeds.

## Introduction

Herbicide-resistant genetically modified (GM) oilseed rape (OSR, *Brassica napus* L.) is cultivated on a large scale in Canada, USA, Chile and Australia [1]. In Canada, one of the world's major producers, the acreage of GM OSR was estimated to be far over 90% of total OSR acreage in 2013. Other countries, especially in Europe, oppose to the cultivation of GM OSR for various reasons. One of the major concerns is the escape of transgenes into the genomes of wild related species, possibly leading to the emergence of transgenic weeds [2], [3]. For OSR, hybridization potential with other Brassicacean species has been extensively investigated [4]-[7]. OSR has a comparatively large number of relatives that occur in agroecosystems with which crosses are possible. Although species hybrids and their backcrosses to parental species generally show reduced fitness, transgenic traits may enhance plant fitness under favourable conditions. Selection pressure provided by herbicide application increases the survival of herbicide-resistant transgenic plants potentially leading to an introgression of transgenes into related species [8], [9]. Another concern with respect to the cultivation of GM OSR is an unintended gene flow towards conventional or organic OSR crops which could lead to co-existence conflicts between different farming systems [10]. In the European Union, GM OSR cultivation is presently prohibited and authorization for the import for food and feed processing is confined to the GM OSR events GT73 (Roundup Ready, Monsanto), MS8, RF3, MS8xRF3 and T45 (all traded as InVigor, Bayer CropScience) [11]. GM crop plants have found even less acceptance in Switzerland where currently neither the import nor the cultivation of GM OSR is allowed at least until the end of 2017 [12], [13]. Nevertheless, the spread of GM OSR cannot totally be prevented by cultivation or import bans. In Japan, where GM OSR is imported but not cultivated, feral glyphosate- and glufosinate-resistant GM OSR plants have repeatedly been detected in port areas and along transportation routes [14]-[17]. The feral GM plants found most likely originated from imported transgenic seeds that were spilled during transport to oilseed processing facilities. Two countrywide studies from Switzerland have reported the occurrence of glyphosate-resistant GT73 OSR from four sites in 2011 and 2012 [18], [19]. The case of Switzerland is remarkable, because GM OSR has neither been cultivated nor imported into the country. The two most affected sites were the Rhine port of Basel and the St. Johann freight railway station in Basel, where seed cargo is regularly imported or was transported in the past, respectively. The feral GT73 OSR probably originated from spillage of conventional OSR seeds or other seed imports that were contaminated with GM seeds [19]. At both sites, vegetation growth is controlled by regular glyphosate treatments. The selective pressure by glyphosate promotes growth of GT73 OSR and increases the escape risk of glyphosate-resistance transgenes through hybridisation and introgression into related species.

The presence of GT73 OSR and the selection pressure in favour of glyphosate-resistance transgenes prompted us to monitor the distribution of feral GM OSR and possible transgene flow from GM OSR to feral non-GM OSR and related plant species in more detail in the Rhine port of Basel and the St. Johann freight railway station. To this end, feral OSR plants and closely related species were sampled in monthly intervals in 2013. Samples were screened for molecular markers of genetical modifications and flow cytometrical analyses were applied to measure nuclear DNA contents of putative species hybrids.

## Materials and Methods

### Sampling sites

The sampling was carried out in the Rhine port of Basel (7°35′26″E, 47°35′12″N) and at the St. Johann freight railway station (7°34′18″E, 47°34′16″N) in Basel.

In the Rhine port of Basel, seed cargo is unloaded from ships and transferred to silos with cranes and finally loaded into railway waggons or lorries for further transport. Based on previous findings of GT73 OSR at two unloading sites [19], we defined high risk areas. These included unloading locations of seed cargo and the railway lines leading to and from them within the Rhine port of Basel. Furthermore, we monitored a perimeter of 100 m adjacent to the high risk areas, which mainly included roads, industrial areas and green areas (embankments, roadside grass verges and fallow land). The perimeter was chosen based on previous field data showing that the probability of cross-pollinations between OSR plants sinks below 1% at a distance of 100 m [20].

At the St. Johann freight railway station, a GT73 OSR population was found at one location on a siding in 2012 [18], [19]. Today, no seed cargo is handled in the St. Johann station, but seed cargo was loaded to freight waggons from silos in 900 m distance and transported through the freight railway station until about 2009. The silos were pulled down and the former loading area was overbuilt. We defined a high risk area that covered railway lines within the St. Johann station and leading away from it. As in the Rhine port of Basel, a perimeter of 100 m adjacent to the high risk area including mainly roads and industrial and green areas was sampled.

At both sampling sites, railway lines are regularly treated with glyphosate. In 2013, spraying was carried out on April 25th, July 25th and October 8th in the Rhine port of Basel and on April 29th at the St. Johann station.

With the exception of peripheral roads and green areas, both sampling sites mainly consisted of railway lines and industrial zones with restricted access. As an enforcement authority, we are generally entitled to access premises for inspections. We received permits to access the Rhine port of Basel and the St. Johann freight railway station by Schweizerische Rheinhäfen (Basel) and Swiss Federal Railways (Bern), respectively.

### Sampling procedure

Plant samples consisted of leaves or entire plants, depending on plant size. Samples were stored in plastic bags and their locations were drawn on a map. If plants had seed pods we collected a subsample of five pods (or all pods, if there were five or less). In the rare case of plants carrying more than 50 pods, ten pods were collected. If several plants were growing together within a distance of 10 m, samples were combined in a pool sample of up to 12 plants. In case of clumped plant assemblies (densities of > 30 plants/m^2^), the total number was estimated and a subsample of 10% of all plants (yet at least 10 plants) was taken. Prior to a sampling round, the positions of already sampled plants were denoted on a map to avoid double sampling. However, at locations with high plant densities it was not always possible to recognize sampled plants with certainty. Therefore, double sampling of plants cannot be ruled out completely at high density locations. If plants with seed pods were still present during a subsequent sampling round, another pod sample was taken.

**Figure 1:**
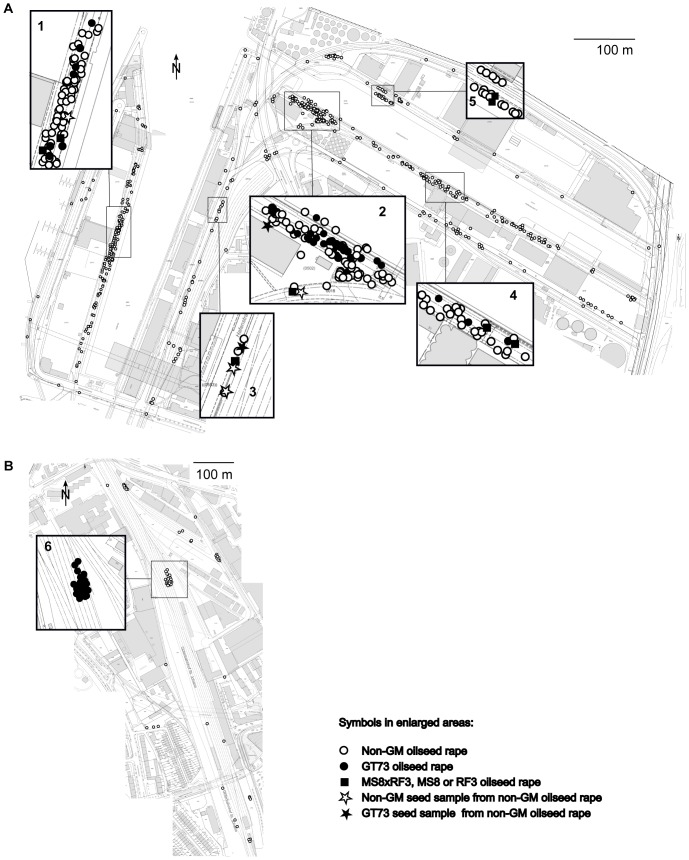
Sample locations in the Rhine port of Basel and the St. Johann freight railway station. Locations of plant samples taken in the Rhine port of Basel (A) and the St. Johann freight railway station (B) are denoted with circles. Locations where genetically modified (GM) oilseed rape (OSR) was found are framed and enlarged. GT73 OSR was found in a previous study at locations 1, 2 (Rhine port) and 6 (St. Johann station). Seed cargo is regularly handled at locations 1, 2, 4 and 5.

OSR seeds have no primary dormancy and may germinate throughout the growing season if conditions are suitable [21], [22]. Therefore, sampling was carried out in intervals of four to five weeks from spring to autumn. In the Rhine port of Basel, plants were sampled on seven dates in 2013. Samplings 1 to 7 were carried out on April 22nd, May 28th, July 2nd, July 29th, September 2nd, October 7th and November 4^th^, respectively. An extra sampling in a limited area was carried out on June 18th due to the finding of RF3 OSR in an embankment (Fig. 1A, location 5). The St. Johann station was sampled on six dates in 2013. Samplings 1 to 6 were conducted on April 25th, June 3rd, July 8th, August 13th, September 10th and October 14th. The St. Johann station was not sampled in November due to the low numbers of plants found in September and October. Plant sampling did not require specific permissions and we did not sample any protected or endangered species.

### DNA extraction

Leaf samples were extracted in pools of up to 12 plants where applicable. For pooled sample analysis, a leaf disc (ø  =  10 mm) was stamped with a hole puncher from each sample. The remaining sample material was frozen and stored at −80°C. Pooled leaf discs or material of single plants were ground to powder in liquid nitrogen using mortar and pestle. Seeds of up to five seed pods of single OSR plants were ground in the same way. In the case of seed samples of either *Sinapis arvensis* L. or *Diplotaxis tenuifolia* (L.) DC., which both have pods with less and smaller seeds than OSR, up to 10 pods were pooled from several individuals growing at the same location to obtain enough seed material for DNA extraction. DNA was extracted from ground samples with the DNeasy Plant Mini Kit (Qiagen) in combination with the extraction robot QIAcube (Qiagen) according to the manufacturer's standard protocol. DNA concentration was measured at 260 nm with a spectrophotometer (Nanodrop ND-1000, Fisher Scientific AG, Switzerland) and diluted to a concentration of 2 ng µl^−1^ for real-time PCR. If pooled leaf samples tested positive for genetic modification markers, DNA was extracted from the frozen samples of each individual plant of the respective pool sample for further analysis.

### Real-time PCR for amplification of genetic modification markers

All DNA samples were amplified and analysed in duplicate on a Rotor-Gene Q real-time PCR cycler (Qiagen) under the following cyling conditions: initial heating for 15 min at 95°C followed by 45 cycles of amplification of 15 s at 95°C and 60 s at 60°C. Real-time PCR was carried out in 25 µl reaction volume containing 1× QuantiTect Multiplex PCR Master Mix (Qiagen), 10 ng template DNA, and the primers and probes specified below. Primers and probes used for the amplification of the plant-specific actin gene and the false negative control (FNC) fragment in a duplex real-time PCR: act-f (0.13 µM), act-r (0.13 µM), act-p (0.035 µM) [23], fnc-f (0.13 µM), fnc-r (0.13 µM), fnc-p (0.035 µM). Primers and probes used for the amplification of the phosphinotricin acetyltransferase genes bar and pat from *Streptomyces hygroscopicus* and *S. viridochromogenes*, respectively, conferring a resistance against the herbicide glufosinate, the glyphosate oxidoreductase gene gox from *Ochrobactrum anthropi* and the enolpyruvylshikimate-3-phosphate synthase gene CP4 epsps from *Agrobacterium tumefaciens* CP4 mediating glyphosate-resistance, in a tetraplex real-time PCR: bar-f (0.4 µM); bar-r (0.4 µM), bar-p (0.2 µM) for the amplification of a 110 bp fragment of the bar gene (GenBank Acc.Nr. X05822.1: 222 - 331); pat-f (0.5 µM), pat-r (0.5 µM), pat-p (0.2 µM) [24]; gox-f (0.5 µM), gox-r (0.5 µM), gox-p (0.2 µM) for the amplification of a 107 bp fragment of the gox gene (Acc.Nr. BD008400, based on a conventional PCR reaction [25]); epsps-f (0.6 µM), epsps-r (0.6 µM), epsps-p (0.2 µM) [24]. Primers and probes used for the detection of the cauliflower mosaic virus 35S promoter (35S-P) and the nopaline synthase terminator (NOS-T) from *A. tumefaciens* in a duplex real-time PCR: 35S-f (0.64 µM), 35S-r (0.64 µM), 35S-p (0.16 µM) [26]; NOS-f (0.64 µM), NOS-r (0.64 µM), NOS-p (0.16 µM) [26]. Primers and probe used for the amplification of the event-specific sequence of GT73: GT73F1 (0.3 µM), GT73R1 (0.9 µM), GT73TMP1 (0.2 µM). Primers and probe used for the amplification of the event-specific sequence of MS8: KVM085 (0.4 µM), HCA048 (0.4 µM), TM011 (0.2 µM) [27]. Primers and probe used for the amplification of the event-specific sequence of RF3: KVM084 (0.4 µM), DPA165 (0.4 µM), TM010 (0.2 µM) [27]. All Primer and probe sequences are presented in Table S1.

DNA samples were first amplified with primers and probes for the plant-specific actin gene and the FNC fragment to test for DNA quality and PCR inhibiting agents. Samples were then amplified with primers and probes for the genes bar, pat, gox, and CP4 epsps, and 35S-P and NOS-T. If samples tested positive for genetic modification markers, they were amplified with primers and probes for the event-specific sequences of GT73, MS8 or RF3.

All primers and probes which were developed in-house were designed using Primer Express Software (Applied Biosystems) and tested in silico for specificity and cross-reactivity using blast from the National Center for Biotechnology Information [28]. All primers and probes were purchased from Eurogentec (Belgium) except primers and probes for the actin gene and the FNC fragment, which were purchased from Microsynth (Switzerland).

### Reference DNA

As positive control for real-time PCR, reference plasmids were used containing the target sequences for the detection of either actin, bar, pat, gox and CP4 epsps or 35S-P and NOS-T as described by Hecht et al. [19]. A plasmid containing a random synthetic DNA sequence of 111 bp length served as FNC [19]. For the detection of the transgenic events GT73, MS8 and RF3, we used genomic DNA as positive control (GMO Genomic DNA Standard Set, Sigma-Aldrich, Switzerland).

### Flow cytometry

Flow cytometry was used to estimate DNA contents of cell nuclei of putative species hybrids and plants that could not be identified with certainty due to small size or due to the absence of flowers or fruits. As putative species hybrids we considered OSR plants with unusual leaf shape, colour or growth form and plants that were identified as related *Brassica* species. Common *Brassica* species and frequent related species were used as references (Table 1). Reference samples were obtained from seed vendors and a genebank. As a reference standard for the estimation of nuclear DNA contents we used *Pisum sativum* L. [29], [30].

**Table 1:** Reference plants used for flow cytometrical analyses.

Species name	Cultivar or Accession-Nr.	Common name	Source
*Brassica carinata* A. Braun.	Carina	Abyssinian mustard	1
*Brassica carinata* A. Braun.	Gomenzer	Abyssinian mustard	1
*Brassica juncea* (L.) Czern.	Green in Snow	Brown mustard, rai	^2^
*Brassica juncea* (L.) Czern.	–	Brown mustard, rai	^3^
*Brassica napus* L. var. *napus*	Heros	Oilseed rape	^4^
*Brassica napus* L. var. *napus*	Elektra	Oilseed rape	^5^
*Brassica napus* L. var. *napobrassica*	Gelbe Schmalz	Rutabaga, swede	^2^
*Brassica nigra* (L.) W.D.J. Koch ssp. *nigra*	CR2713	Black mustard	^6^
*Brassica nigra* (L.) W.D.J. Koch ssp. *nigra*	CR2734	Black mustard	^6^
*Brassica oleracea* L. var. *capitata*	Holsteiner Platter	Cabbage	^2^
*Brassica oleracea* L. var. *capitata*	Plainpalais à pied court	Savoy cabbage	^2^
*Brassica oleracea* L. var. *gongylodes*	Delikatess Weisser	Kohlrabi	^2^
*Brassica rapa* L. ssp. *rapa*	Zürcher Stoppelrübe	Turnip	^2^
*Brassica rapa* L. ssp. *rapa*	Navet de Croissy	Turnip	^2^
*Brassica rapa* L. var. *cymosa*	–	Cima di rapa	^2^
*Diplotaxis tenuifolia* (L.) DC.	–	Perennial wall-rocket	^2^
*Eruca sativa* Mill.	–	Salad rocket	1
*Sinapis alba* L.	–	Yellow or white mustard	^2^
*Sinapis arvensis* L.	–	Charlock	^7^

1 Rühlemann's, Horstedt, Germany; ^2^ Botanik Sämereien, Zürich, Switzerland; ^3^ Templiner Kräutergarten, Templin, Germany; ^4^ FiBL, Frick, Switzerland; ^5^ Landi Reba, Basel, Switzerland; ^6^ Genbank IPK Gatersleben, Stadt Seeland, Germany; ^7^ fenaco, Winterthur, Switzerland.

Leaves of sampled plants and of *P. sativum* Feltham First were chopped together with a sharp razor blade in a Petri dish containing 0.4 ml extraction buffer (CyStain UV Precise P Nuclei Extraction Buffer, Partec, Switzerland) for 1 min. After 1 min of incubation the solution was filtrated through a 50 µm CellTrics filter (Partec) and 1.6 ml of 4′,6-diamidino-2-phenylindole (DAPI) staining buffer (CyStain UV Precise P Nuclei Staining Buffer, Partec, Switzerland) was added. After 3 min of staining, fluorescence intensities of nuclei were measured with a CyFlow Ploidy Analyzer (Partec, Switzerland) equipped with a UV-LED of 365 nm emission wavelength. Data were analysed with Flowing Software (Cell Imaging Core, Turku Centre for Biotechnology, Finland). Relative 2C DNA content values, i.e. the DNA content values of unreplicated diplophasic nuclei [30], were calculated by dividing the 2C DNA content value of the sample through the 2C DNA content value of the reference standard. For reference plant samples and reference standard only fresh young leaves were used. For field plant samples, the youngest leaves available were used. Reference samples were measured at least three times, field samples were measured once.

Single measurements of 2C DNA content values (hereafter called 2C values) of reference plants had coefficients of variation (CV) below 5% except some samples of *Brassica nigra* (L.) W.D.J. Koch, *Brassica oleracea* L., *Brassica rapa* L. and *D. tenuifolia* (CV ≤ 7%). Reference samples of different *Brassica* species could be clearly distinguished on the basis of 2C values (Table 2). Although 2C values of *Brassica* and related species were sometimes similar (e.g. the 2C values of *B. nigra* and *S. arvensis*), the 2C value of OSR was well distinguishable.

**Table 2:** Relative 2C DNA contents of cell nuclei of *Brassica napus* and related species sorted by value.

Plant species and cultivar or accession number	Relative 2C DNA content (± SD)
*B. carinata* Carina	0.316 ± 0.002
*B. carinata* Gomenzer	0.316 ± 0.001
*B. napus* var. *napus* Elektra	0.301 ± 0.002
*B. napus* var. *napus* Heros	0.298 ± 0.004
*B. napus* var. *napobrassica*	0.295 ± 0.006
*B. juncea*	0.270 ± 0.001
*B. juncea* Green in Snow	0.266 ± 0.002
*Diplotaxis tenuifolia*	0.206 ± 0.004
*Eruca sativa*	0.182 ± 0.004
*B. oleracea* var. *capitata*	0.175 ± 0.001
*B. oleracea* var. *gongylodes*	0.172 ± 0.002
*B. oleracea* var. *sabauda*	0.172 ± 0.001
*B. nigra* CR2734	0.153 ± 0.006
*B. nigra* CR2713	0.150 ± 0.003
*Sinapis arvensis*	0.144 ± 0.002
*S. alba*	0.133 ± 0.001
*B. rapa* ssp. *rapa* Navet	0.130 ± 0.002
*B. rapa* ssp. *rapa* Zürcher	0.129 ± 0.003
*B. rapa* var. *cymosa*	0.127 ± 0.001

Relative 2C DNA contents were calculated with *Pisum sativum* Feltham First as standard. All samples were measured three times except *B. napus* var. *napus* Heros, which was measured 12 times.

2C values of field plant samples were compared to 2C values of reference plant samples to determine their species.

### Real-time PCR for amplification of a genome specific *Brassica oleracea* L. microsatellite

Some field samples that were assigned to the species *Brassica juncea* (L.) Czern showed slightly higher 2C values than *B. juncea* reference samples in flow cytometrical analyses (see results section). To test for putative hybrid identity of field samples, we carried out a PCR to amplify the microsatellite marker 83b1 in four samples with high 2C values [31]. Microsatellite 83b1 is present in the genome of *B. oleracea*, one of the two ancestor species of *B. napus* [32] and has been used repeatedly to identify hybrids between *B. napus* and other *Brassica* species [32]-[34].

Template DNA was amplified as described above on a Rotor-Gene Q real-time PCR cycler (Qiagen) with primers 83b1-f (0.64 µM) and 83b1-r (0.64 µM) (Table S1). PCR products were examined on a 1% agarose gel stained with ethidium bromide.

## Results

### Relatives of OSR

Species and sample numbers of relatives of OSR differed between sampling sites and dates (Table S2). In the Rhine port of Basel, the wild relative *D. tenuifolia* was very common, especially in embankments.The high plant numbers and the inconspicuous growth form of *D. tenuifolia* made it impractical to reliably estimate the size of the total population. We sampled 105 plants of which 15 had seed pods. *Sinapis arvensis* was relatively common and 77 samples were taken of which 11 had seed pods.The only *Brassica* species found was *B. juncea* (15 samples), which grew at different locations as individuals or in small groups. Furthermore, three *Rapistrum rugosum* (L.) All. plants were sampled. In the St. Johann station, relatives of OSR were less abundant. We sampled 12 *D. tenuifolia* and a single *S. arvensis* plant. Leaf and seed pod samples were collected from four *B. oleracea* plants in a family garden in 750 m distance of the known GT73 OSR site.

None of the leaf or seed pod samples of *D. tenuifolia*, *S. arvensis*, *B. juncea*, *B. oleracea* and *R. rugosum* contained genetic modification markers.

### OSR

In the Rhine port of Basel, 2291 OSR plants were sampled of which 61 had seed pods. OSR often grew in patches of high density which resulted in a large number of subsamples. The total estimated number of OSR plants present at all sampling dates amounted to 4863.

In the St. Johann station, we sampled 279 OSR plants of which 28 had seed pods. OSR plants were growing in low density except at the known GT73 OSR location. The total estimated number of OSR plants present at all sampling dates amounted to 418.

**Figure 2:**
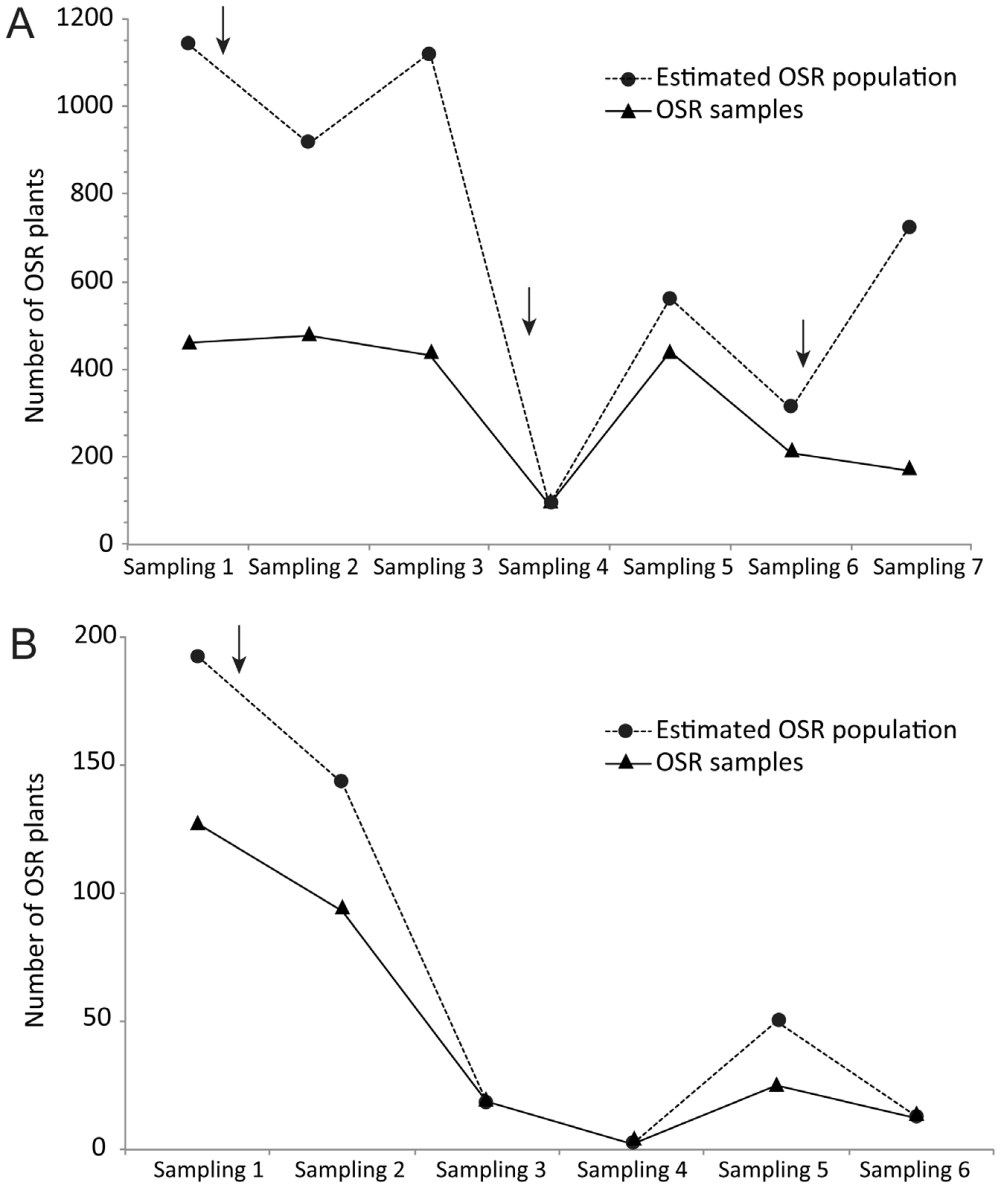
Oilseed rape (OSR) in the Rhine port of Basel and the St. Johann freight railway station. Estimated total OSR population and OSR sample numbers taken at the different sampling dates in the Rhine port of Basel (A) and the St. Johann freight railway station (B). Arrows indicate glyphosate treatment of railway lines.

**Figure 3:**
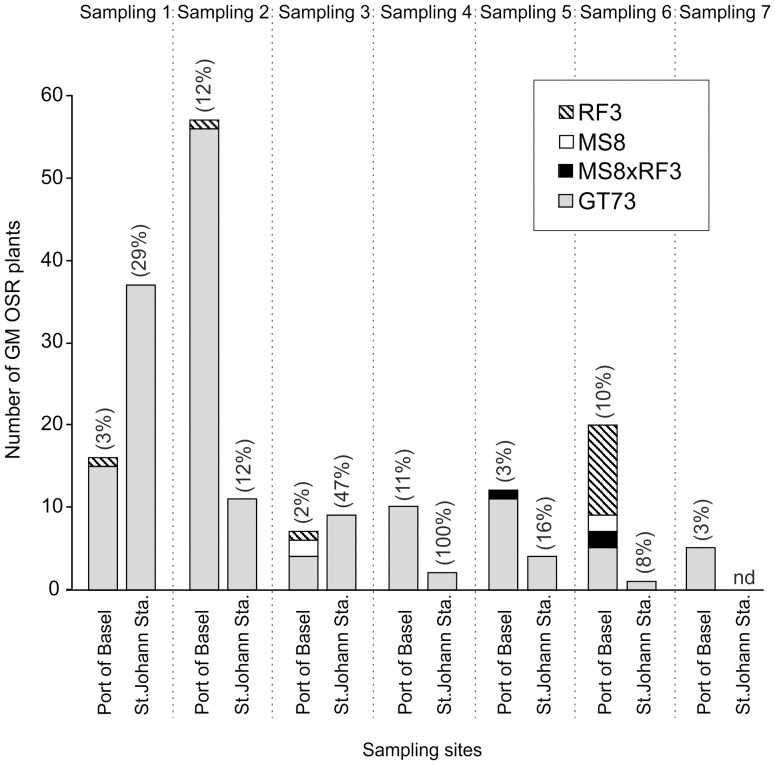
Genetically modified (GM) oilseed rape (OSR) in the Rhine port of Basel and the St. Johann freight railway station. Numbers of GM OSR events MS8, RF3, MS8xRF3 and GT73 at the different sampling dates in the Rhine port of Basel and the St. Johann freight railway station. Percentage values in brackets indicate the proportion of GM OSR plants in the total OSR sample.

Differences in OSR plant numbers between dates were pronounced at both sites (Fig. 2). Numbers of GM OSR plants found at the two sites on different sampling dates are presented in Figure 3. In the Rhine port of Basel, 106 out of 2291 OSR leaf samples contained the transgenes gox and CP4 epsps that confer resistance to glyphosate as well as the event-specific DNA sequence of transgenic GT73 OSR. Presence of GT73 OSR plants could be reconfirmed at the two already known locations (Fig. 1A; locations 1 and 2) and was detected at one new location (Fig. 1A; location 4). Furthermore, 22 OSR leaf samples tested positive for the bar gene conferring resistance to glufosinate. Thereof, 4, 13 and 3 plants contained the event-specific DNA sequence of transgenic MS8, RF3 or MS8 and RF3 OSR, respectively. MS8xRF3, MS8 or RF3 OSR was detected for the first time at five locations in the port area (Fig. 1A; locations 1–5). At three of these locations, GM OSR was found for the first time (Fig. 1A; locations 3–5). Transgenic MS8xRF3 OSR is a hybrid variety bred from the male sterile line MS8 and the fertility restoration line RF3 [35] and seeds produced by MS8xRF3 OSR can carry either one or both event-specific sequences of MS8 and RF3. Although GT73 OSR was the most prevalent GM OSR event in the Rhine port of Basel, it was only found at three locations. MS8xRF3, MS8 or RF3 plants were less frequent than GT73 but had a wider distribution, growing at five locations. GM OSR plants showed an aggregated distribution at unloading sites (Fig. 1A; locations 1, 2, 4, 5). Only one place of discovery was located between a road and railway lines leading to and out of the Rhine port of Basel (Fig. 1A, location 3).Two out of 48 seed pod samples from non-GM OSR mother plants contained the transgenes gox and CP4 epsps as well as the event-specific DNA sequence of transgenic GT73 OSR, indicating at least partial pollination of flowers with GT73 OSR pollen. The first seed pod sample was collected in immediate vicinity of GT73 OSR plants (Fig. 1A, location 2), the second was sampled 200 m away from the nearest detected GT73 plant (Fig. 1A, location 3).

In the St. Johann station, 64 out of 279 OSR leaf samples contained the transgenes gox and CP4 epsps as well as the event-specific DNA sequence of transgenic GT73 OSR. Presence of GT73 OSR was reconfirmed at the known location (Fig. 1B, location 6). No new locations with GT73 OSR were detected.

### Species identification by flow cytometry and microsatellite analysis

We measured 2C values of 43 field plants, which produced CV values of up to 8% (Table S3). Based on 2C values and morphological characters, 33 samples could be assigned to *B. napus* (7 plants), *D. tenuifolia* (12 plants) or *S. arvensis* (14 plants) (Table S3).

Ten plant samples yielded 2C values from 0.260 to 0.286 (Table S3), that were scattered around the mean *B. juncea* reference 2C value of 0.268 (Table 2). The higher of these 2C values were in between *B. napus* and *B. juncea* reference 2C values and could be expected for hybrids between the two species (Table 2). However, amplification of the *B. napus*-specific microsatellite marker 83b1 in these plant samples yielded no PCR-product (data not shown) and all samples in question were assigned to *B. juncea*.

## Discussion

Sampling of the St. Johann station and the Rhine port of Basel showed different dynamics of GM OSR spread with new locations and new GM OSR events detected only in the Rhine port of Basel. The discovery of new locations may not indicate an actual spread of GM plants, but may have resulted from the intensified sampling in 2013 compared to previous samplings [19]. To our knowledge, our finding of feral OSR plants containing the GM OSR events MS8 and/or RF3 in the Rhine port of Basel represents the first published finding in Europe.

The considerable differences in GM OSR distribution in the Rhine port of Basel and the St. Johann station are correlated with the extent of seed handling. There is no current transport or loading of seed cargo in the St. Johann station and introduction of seeds through transports on the passing main railway line would probably be rare. Because seed cargo used to be transported from former silos through the St. Johann station until about 2009, the present GT73 OSR population probably has originated from this period. In contrast, regular unloading and transport of seed shipments takes place in the Rhine port of Basel resulting in a constant introduction of seeds through spillage. There, the large population of feral OSR provides opportunities for the spread of transgenes through intraspecific outcrossing of GM OSR. Although the contribution of self-recruitment via seeds of GM and non-GM OSR compared to recruitment from spilled import seeds is unknown, the double finding of transgenic seeds from non-GM OSR plants indicates that this pathway may play a role in transgene spread. In comparison, the small numbers and discontinuous distribution of OSR surrounding the GM OSR population in the St. Johann station limit outcrossing opportunities.

Concerning the risk of interspecific outcrossing of GM OSR, *B. juncea* was the only species found with high potential for hybridization with OSR [6]. However, it is an adventitious species in Switzerland and not common in the area [36]. The *B. juncea* plants found in the Rhine port of Basel very likely have originated from seed imports and do not represent a stepping stone for the spread of transgenes into wild *B. juncea* populations. The hybridization potential of all the other species found has been rated low (*B. oleracea*, *S. arvensis*) to extremely low (*D. tenuifolia*, *R. rugosum*) [6]. Although other species with high hybridization potential (*B. rapa*, *Hirschfeldia incana* (L.) Lagr.-Foss.) have previously been reported for the Rhine port of Basel and the St. Johann station [36], they were not detected in that sampling. Due to the small numbers of species with high hybridization potential the risk of transgene escape through hybridization and introgression into related species seems to be low at both sites. However, the regular application of glyphosate herbicide on railway lines may promote the establishment of species hybrids with a transgenic trait for glyphosate-resistance and further monitoring of wild relatives is recommended.

The aggregated distribution of GM OSR plants at unloading sites in the Rhine port of Basel indicates that GM OSR was introduced during unloading of seed shipments. Although no GM OSR is imported into Switzerland, contaminations with GM OSR events, that are approved in the European Union, are tolerated up to a proportion of 0.5% in OSR imports [37], [38]. However, enquiries at the operating companies showed that there was no import of OSR seed during 2012 and 2013 with the exception of a few hundred kg of crushed OSR. Although small proportions of crushed OSR may still be germinable, it seems more likely that other seed goods, which were contaminated with GM OSR, were the source of introduction of GM OSR. In the Rhine port of Basel, wheat is the major imported seed good. From 2010 to 2012, Switzerland imported 247’000 t of Canadian wheat, accounting for one fifth of its total wheat imports [39]–[41]. As nearly all OSR grown in Canada is GM and cereals are the most frequent used crops following OSR in a typical rotation [42], it is likely that GM OSR is introduced into Switzerland as a contaminant of Canadian wheat imports. However, this pathway is yet to be confirmed. To date, wheat imports into Switzerland are not routinely tested for GM contents, since there is no cultivation of GM wheat worldwide. To further elucidate introduction pathways of GM OSR seeds in the Rhine port of Basel, analyses of imported seed shipments are required.

A quantitative assessment of the amounts of GM and non-GM OSR seeds that are accidentally introduced through seed imports is not possible on the basis of field sample numbers. Since it is not known for how long GM OSR has been present in the St. Johann and the Rhine port of Basel, we cannot deduce whether the actual GM populations mainly originated from self-recruitment of an unknown number of founder plants or whether they are sustained by continuous introduction of GM OSR seeds. OSR is an early successional annual or biennial plant, which grows well on disturbed sites [6]. Studies on the population dynamics of feral OSR along road verges and field margins showed that a large part of OSR populations is transient and occupies a patch for only 1 or 2 years before local extinction [43]–[45]. However, genetic analyses showed moderate to very high differentiation between commercial varieties and feral OSR populations in Central Europe indicating that feral OSR is able to maintain persistent self-recruiting populations [46]. Furthermore, OSR seeds can persist for several years in the soil. Data from agricultural fields showed that OSR volunteers originated from varieties cultivated 4 to 17 years earlier [47]. In another case, GM OSR volunteers were found 10 years after a field trial of herbicide-resistant GM OSR, although volunteer control was strict and no selecting herbicides were applied after the field trial was completed [48]. In the St. Johann station and the Rhine port of Basel, there are large open gravel areas along the railway lines, which are kept free of vegetation by spraying of glyphosate. Regarding habitat requirements and given the potential for seed persistence, both sites provide favourable conditions for self-recruitment of feral glyphosate-resistant GM OSR. Therefore, long-term persistence of glyphosate-resistant GM OSR seems very likely at both sites. Glufosinate-resistant GM OSR was also present at three locations in the port area where glyphosate is regularly applied suggesting that either glufosinate-resistant GM OSR is introduced on a regular basis or that glyphosate spraying is not always carried out efficiently or timed properly.

Notwithstanding ecological considerations, an efficient control of glyphosate-resistant GM OSR along railway lines is in the interest of operating companies for economic reasons. The main purposes of vegetation control on railway lines are to ensure worker safety and stability of the railway gravel bed. In Switzerland, glyphosate is the only authorised herbicidal agent for railway lines [49]. Thus, under the current herbicide directive, further spread of glyphosate-resistant GM OSR would necessitate increased manual vegetation control measures along railway lines.

In conclusion, our study points towards the important role of seed import by ships for the accidental introduction of GM OSR seeds into Switzerland. Future efforts to contain or to eradicate feral GM OSR should be adapted accordingly. Regarding the studied sites, the eradication of feral GM OSR appears achievable in the St. Johann station through continuous manual control of plants, as a new introduction of GM OSR seeds seems unlikely. However, at the Rhine port of Basel a containment plan would have to include measures to prevent a continuous introduction of GM OSR seeds. We suggest an extended testing of import seed cargo to identify contamination sources and recommend minimizing the spillage of seed cargo during unloading and subsequent transport.

## Supporting Information

Table S1 **Specifications of primer and probe systems.**
(DOCX)

Table S2 **Sample numbers of relatives of oilseed rape (** ***Brassica napus*** **) taken at different sampling dates in the Rhine port of Basel and the St. Johann freight railway station.**
(DOCX)

Table S3 **Relative 2C DNA contents of putative hybrids and field plant samples that were difficult to identify by morphological characters.**
(DOCX)
