# Supplementary material for: Unexpected Diversity of Feral Genetically Modified Oilseed Rape (Brassica napus L.) Despite a Cultivation and Import Ban in Switzerland
Source: PLoS One. 2014 Dec 2;9(12):e114477. doi: 10.1371/journal.pone.0114477 (PMC4252112; doi:10.1371/journal.pone.0114477)
Supplement: Table S1 — Specifications of primer and probe systems. (DOCX) [file pone.0114477.s001.docx]

**Table S1** Specifications of primer and probe systems.

| **Gene or target sequence** | **Oligonucleotide** | **Name** | **Sequence 5‘-3‘** | **Reference** |
| --- | --- | --- | --- | --- |
| actin | Forward primer  Reverse primer  Probe | act-f  act-r  act-p | CAA GCA GCA TGA AGA TCA AGG T  ACA ATC TGT TGG AAA GTG CT GAG  ROX-CCT CCA ATC CAG ACA CTG TAC TTY CTC TC-BHQ2 | [[23](#_ENREF_22)] |
| False negative control | Forward primer  Reverse primer  Probe | fnc-f  fnc-r  fnc-p | CGT CAC ATC GGT AGA CGA ACT AA  TTC AAG TCC TGA GCG GTT GTA A  JOE-ACC TAA CGC AGC AAC TTA TCG ACC GTT CAC TT-BHQ1 | In-house |
| 35S-P | Forward primer  Reverse primer  Probe | 35S-f  35S-r  35S-p | GCC TCT GCC GAC AGT GGT  AAG ACG TGG TTG GAA CGT CTT C  FAM-CAA AGA TGG ACC CCC ACC CAC G-BHQ1 | [[26](#_ENREF_25)] |
| NOS-T | Forward primer  Reverse primer  Probe | NOS-f  NOS-r  NOS-p | ATG ACG TTA TTT ATG AGA TGG GTT TTT A  TTG CGC GCT ATA TTT TGT TTT C  YY-AGA GTC CCG CAA TTA TAC ATT TAA TAC GCG A-BHQ1 | [[26](#_ENREF_25)] |
| bar | Forward primer  Reverse primer  Probe | bar-f  bar-r  bar-p | CTG CAC CAT CGT CAA CCA CTA C  GAT AGC GCT CCC GCA GAC  FAM-CGT ACC GAG CCG CAG GAA CCG CAG GAG T-BHQ1 | In-house |
| pat | Forward primer  Reverse primer  Probe | pat-f  pat-r  pat-p | CGC GGT TTG TGA TAT CGT TAA C  TCT TGC AAC CTC TCT AGA TCA TCA A  CY5-AGG ACA GAG CCA CAA ACA CCA CAA GAG TG-BHQ2 | [[24](#_ENREF_23)] |
| CP4 epsps | Forward primer  Reverse primer  Probe | epsps-f  epsps-r  epsps-p | CCA ATG GGT CGT GTG TTG AA  TTG GCG TTG GAG TCT TTG GT  JOE-AGA CGG TGA TCG TCT TCC AGT TAC CTT GC-BHQ1 | [[24](#_ENREF_23)] |
| gox | Forward primer  Reverse primer  Probe | gox-f  gox-r  gox-p | CCG TGG AGG TTG GGA ACT T  CCC TTG GTA AAG GCG TGA GA  ROX-CTG ATG CAT TGC GTG ATT TCG ATC CTA AC-BHQ2 | In-house, based on [[25](#_ENREF_24)] |
| GT73 | Forward primer  Reverse primer  Probe | GT73F1  GT73R1  GT73TMP1 | TCA TAC TCA TTG CTG ATC CAT GTA GA  AAG CTT ATA CGA AGG CAA GAA AAG G  FAM-TTC CCG GAC ATG AAG ATC ATC CTC CTT C-DABCYL | In-house |
| MS8 | Forward primer  Reverse primer  Probe | KVM085  HCA048  TM011 | GTT AGA AAA AGT AAA CAA TTA ATA TAG CCG G  GGA GGG TGT TTT TGG TTA TC  FAM-AAT ATA ATC GAC GGA TCC CCG GGA ATT C-TAMRA | [[27](#_ENREF_26)] |
| RF3 | Forward primer  Reverse primer  Probe | KVM084  DPA165  TM010 | AGC ATT TAG CAT GTA CCA TCA GAC A  CAT AAA GGA AGA TGG AGA CTT GAG  FAM-CGC ACG CTT ATC GAC CAT AAG CCC A-TAMRA | [[27](#_ENREF_26)] |
| 83b1 | Forward primer  Reverse primer | 83b1-f  83b1-r | GCC TTT CTT CAC AAC TGA TAG CTA A  TCA GGT GCC TCG TTG AGT TC | [[31](#_ENREF_29)] |
