# Supplementary material for: Unexpected Diversity of Feral Genetically Modified Oilseed Rape (Brassica napus L.) Despite a Cultivation and Import Ban in Switzerland
Source: PLoS One. 2014 Dec 2;9(12):e114477. doi: 10.1371/journal.pone.0114477 (PMC4252112; doi:10.1371/journal.pone.0114477)
Supplement: Table S2 — Sample numbers of relatives of oilseed rape ( Brassica napus ) taken at different sampling dates in the Rhine port of Basel and the St. Johann freight railway station. (DOCX) [file pone.0114477.s002.docx]

**Table S2** Sample numbers of relatives of oilseed rape (*Brassica napus*) taken at different sampling dates in the Rhine port of Basel and the St. Johann freight railway station.

|  | ***B. juncea*** | ***B. oleracea*** | ***Diplotaxis tenuifolia*** | ***Rapistrum rugosum*** | ***Sinapis arvensis*** |
| --- | --- | --- | --- | --- | --- |
| **Rhine port** |  |  |  |  |  |
| April 22nd | - | - | 9 | - | - |
| May 28th | - | - | 14 | - | 42 |
| June 18th* | - | - | 1 | 3 | - |
| July 2nd | 2 | - | 50 | - | 17 |
| July 29th | - | - | 15 | - | - |
| September 2nd | 1 | - | 16 | - | 2 |
| October 7th | 6 | - | - | - | 14 |
| November 4th | 6 | - | - | - | 2 |
| Total | 15 | - | 105 | 3 | 77 |
| **St. Johann station** |  |  |  |  |  |
| April 25th | - | - | - | - | 1 |
| June 3rd | - | - | 8 | - | - |
| July 8th | - | - | 4 | - | - |
| August 13th | - | - | - | - | - |
| September 10th | - | - | - | - | - |
| October 14th | - | 4 | - | - | - |
| Total | - | 4 | 12 | - | 1 |

* Extra sampling carried out in a limited area.
