# Supplementary material for: Unexpected Diversity of Feral Genetically Modified Oilseed Rape (Brassica napus L.) Despite a Cultivation and Import Ban in Switzerland
Source: PLoS One. 2014 Dec 2;9(12):e114477. doi: 10.1371/journal.pone.0114477 (PMC4252112; doi:10.1371/journal.pone.0114477)
Supplement: Table S3 — Relative 2C DNA contents of putative hybrids and field plant samples that were difficult to identify by morphological characters. (DOCX) [file pone.0114477.s003.docx]

**Table S3** Relative 2C DNA contents of putative hybrids and field plant samples that were difficult to identify by morphological characters.

| **Sample-Nr.** | **Relative 2C DNA content** | **Coefficient of variation (%)** | **Identified species** |
| --- | --- | --- | --- |
| 1 | 0.131 | 2.9 | *S. arvensis* |
| 2 | 0.136 | 5.9 | *S. arvensis* |
| 3 | 0.137 | 7.6 | *S. arvensis* |
| 4 | 0.141 | 4.6 | *S. arvensis* |
| 5 | 0.141 | 5.4 | *S. arvensis* |
| 6 | 0.141 | 4.6 | *S. arvensis* |
| 7 | 0.141 | 5.6 | *S. arvensis* |
| 8 | 0.143 | 6.3 | *S. arvensis* |
| 9 | 0.143 | 8.0 | *S. arvensis* |
| 10 | 0.144 | 6.6 | *S. arvensis* |
| 11 | 0.144 | 6.1 | *S. arvensis* |
| 12 | 0.146 | 6.3 | *S. arvensis* |
| 13 | 0.146 | 3.8 | *S. arvensis* |
| 14 | 0.153 | 3.8 | *S. arvensis* |
| 15 | 0.195 | 7.2 | *D. tenuifolia* |
| 16 | 0.196 | 6.0 | *D. tenuifolia* |
| 17 | 0.196 | 4.6 | *D. tenuifolia* |
| 18 | 0.198 | 4.9 | *D. tenuifolia* |
| 19 | 0.198 | 4.0 | *D. tenuifolia* |
| 20 | 0.200 | 4.5 | *D. tenuifolia* |
| 21 | 0.201 | 3.7 | *D. tenuifolia* |
| 22 | 0.201 | 4.6 | *D. tenuifolia* |
| 23 | 0.203 | 6.5 | *D. tenuifolia* |
| 24 | 0.204 | 5.8 | *D. tenuifolia* |
| 25 | 0.207 | 4.6 | *D. tenuifolia* |
| 26 | 0.209 | 4.8 | *D. tenuifolia* |
| 27 | 0.260 | 6.2 | *B. juncea* |
| 28 | 0.261 | 5.2 | *B. juncea* |
| 29 | 0.271 | 4.7 | *B. juncea* |
| 30 | 0.273 | 6.4 | *B. juncea* |
| 31 | 0.275 | 6.1 | *B. juncea* |
| 32 | 0.276 | 3.6 | *B. juncea* |
| 33 | 0.277 | 4.8 | *B. juncea* |
| 34 | 0.280 | 4.8 | *B. juncea* |
| 35 | 0.281 | 4.7 | *B. juncea* |
| 36 | 0.286 | 5.0 | *B. juncea* |
| 37 | 0.290 | 5.8 | *B. napus* |
| 38 | 0.299 | 4.4 | *B. napus* |
| 39 | 0.300 | 4.5 | *B. napus* |
| 40 | 0.301 | 3.4 | *B. napus* |
| 41 | 0.303 | 3.6 | *B. napus* |
| 42 | 0.305 | 4.0 | *B. napus* |
| 43 | 0.306 | 5.5 | *B. napus* |

Based on relative 2C DNA contents of reference samples and morphological characters field samples were identified (mean relative 2C DNA contents of *Sinapis arvensis,* *Diplotaxis tenuifolia,* *Brassica juncea* and *B. napus* reference samples were 0.144, 0.206, 0.268 and 0.298, respectively). Relative 2C DNA contents were calculated with *Pisum sativum* Feltham First as standard. All samples were measured once.
